# 5‐ARI induces autophagy of prostate epithelial cells through suppressing IGF‐1 expression in prostate fibroblasts

**DOI:** 10.1111/cpr.12590

**Published:** 2019-03-18

**Authors:** Bo‐Yu Yang, Chen‐Yi Jiang, Chen‐Yun Dai, Rui‐Zhe Zhao, Xing‐Jie Wang, Yi‐Ping Zhu, Yu‐Xin Qian, Fu‐Li Yin, Xiang‐Yu Fu, Yi‐Feng Jing, Bang‐Min Han, Shu‐Jie Xia, Yuan Ruan

**Affiliations:** ^1^ Department of Urology, Shanghai General Hospital Shanghai Jiao Tong University School of Medicine Shanghai China; ^2^ Institute of Translational Medicine Shanghai General Hospital Shanghai China

**Keywords:** androgen receptor, benign prostatic hyperplasia, insulin‐like growth factor 1, prostate epithelial cells, prostate fibroblasts

## Abstract

**Objectives:**

5α‐reductase inhibitor (5‐ARI) is a commonly used medicine in the treatment of lower urinary tract symptoms (LUTS) associated with benign prostatic hyperplasia (BPH). Our study mainly focuses on the mechanism of BPH development after 5ARI treatment.

**Materials and Methods:**

Prostate specimens from patients were collected. Insulin‐like growth factor 1 (IGF‐1), Beclin‐1, LC3 levels, was analysed by immunohistochemistry. The role IGF‐1 on autophagic flux in prostate epithelial cells was studied. Additionally, effect of autophagy on recombinant grafts consisting of prostate stromal and epithelial cells in nude mice was investigated.

**Results:**

We demonstrated that IGF‐1 expression is down‐regulated in prostate fibroblasts after long‐term 5‐ARI application. A decrease in IGF‐1 levels was found to activate autophagic flux through the mTOR pathway in prostate epithelial cells, while the inhibition of IGF‐1 receptor function induced autophagy in prostate epithelial cells. In addition, we revealed that blocking autophagic flux initiation can reduce the volume of recombinant grafts in vivo. Finally, our findings suggest that long‐term 5‐ARI application reduces IGF‐1 secretion by prostatic stromal cells, thereby inducing autophagy of prostatic epithelial cells, which is one of the mechanisms underlying BPH pathogenesis and progression.

**Conclusions:**

Focusing on the autophagy induced by low levels of IGF‐1 in prostatic epithelial cells, after elucidating AR signalling impairment of prostate stromal cells, might provide a novel strategy for the treatment and prevention of BPH development.

## 1 INTRODUCTION

Benign prostatic hyperplasia (BPH), which is commonly associated with lower urinary tract symptoms (LUTS), is a highly prevalent disease that is characterized by the proliferation of the human prostate and greatly affects the quality of life of ageing men.^1^ The prostate is a hormone‐responsive organ, and androgen receptor (AR) signalling plays a fundamental role in BPH progression. Androgen ablation therapy, such as the use of finasteride, dutasteride and other 5α‐reductase inhibitors (5‐ARIs), can impair AR signalling by inhibiting the production of dihydrotestosterone (DHT).^2^ Reducing androgen levels has been universally recognized as an effective method for treating BPH by inducing prostate epithelial cell apoptosis.^3^ However, clinical progression still occurs in more than 17% of BPH patients who receive long‐term 5‐ARI treatment, and the detailed molecular and cellular mechanisms remain to be determined.^4^, ^5^

The prostate consists of fibroblasts and epithelial cells, and its microenvironment is complex.^6^ Prostate epithelial cells are non‐cell autonomously regulated by cytokines released by prostate fibroblasts in a paracrine manner. Thus, the interaction between fibroblasts and epithelial cells also plays a pivotal role in BPH progression and other prostatic disease.^7^ Our previous study found that the expression of LC3A/B and Beclin‐1 was significantly increased in BPH tissues compared with that in control group prostate tissue.^8^ Autophagy is a highly conserved lysosomal pathway in eukaryotes that functions to recycle and degrade various cellular components.^9^ Autophagy often exerts cytoprotective functions to sustain the re‐establishment of the cell status. That is, the inhibition of autophagy can induce cell apoptosis or death.^10^ Another study has also shown that hypoxia‐induced autophagy promotes the survival of human prostatic cells.^11^ Bennett *et al* found that the blockade of androgen/AR signalling in prostate epithelial cells leads to increased autophagy.^12^ Furthermore, the expression of autophagy‐related genes is higher in prostate epithelial cells after reducing androgen levels.^13^ Thus, we hypothesized that autophagy might lead to prostate epithelial cell proliferation and BPH progression after long‐term 5‐ARI treatment.

## 2 MATERIALS AND METHODS

Materials and methods are described in detail in Data S1.

## 3 RESULTS

### 3.1 The expression of IGF‐1 is down‐regulated in prostate stromal cells after AR signalling impairment

**Figure 1:**
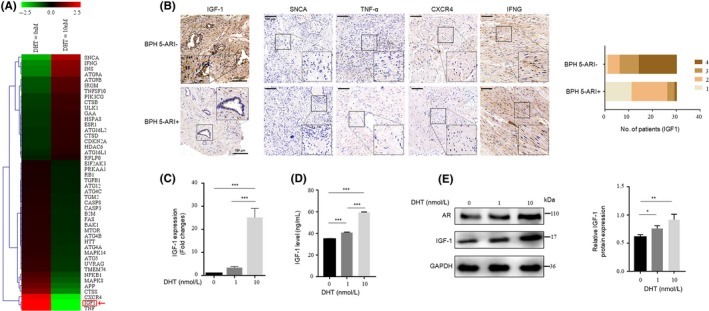
Androgen deprivation reduces IGF‐1 expression in prostate fibroblasts. A, Heatmap showing 89 autophagy‐related differentially expressed genes in WPMY‐1‐AR cells treated with 10 nmol/L DHT. Red arrow pointed to IGF‐1. B, Left, immunohistochemical analysis and statistical graph of the expression levels of IGF‐1, SNCA, TNF, CXCR4 and IFNG in the prostate tissues. Right, statistical graph and analysis of the IHC results of IGF‐1 in the prostate epithelium that were scored semi‐quantitatively as follows: 1: negative; 2: weakly positive; 3: moderately positive; and 4: strongly positive staining (n = 30). (Scale bars, 100 μm). (C‐E) WPMY‐1‐AR cells were treated with 0/1/10 nmol/L DHT after incubation for 48 h with phenol red‐free DMEM without FBS; 24 h later, the mRNA and protein expression levels of IGF‐1 were detected by qRT‐PCR (C) and ELISA (D). Western blotting results (E) showing IGF‐1 and AR protein expression. *P < 0.05, **P < 0.01, ***P < 0.001

Our previous work focused mainly on prostatic epithelial cells that were autonomously regulated by stromal cells, and we performed a qRT‐PCR array analysis to screen genes associated with autophagy after androgen deprivation of prostate stromal cells stably expressing AR (WPMY‐1‐AR cells) (Table S1).^14^ The PCR array analysis showed that the WPMY‐1‐AR cells exhibited more significant changes in their expression levels of several genes when treated with 0 nmol/L DHT than when the cells were treated with 10 nnmol/L DHT (Figure 1A, Figure S1). Genes that were more than 6.5‐fold up‐ or down‐regulated, which included IGF‐1, α‐synuclein (SNCA), tumour necrosis factor (TNF), C‐X‐C chemokine receptor type 4 (CXCR4) and interferon gamma (IFNG), were examined in the prostate specimens of patients. The clinical parameters of the participants in this study were shown in Table S2. However, only IGF‐1 showed an obvious decrease after 5‐ARI treatment (BPH 5‐ARI+versus BPH 5‐ARI‐; Figure 1B, Table S3). To further confirm the effects of different concentrations of DHT on IGF‐1 expression in prostatic stromal cells, we performed qRT‐PCR (Figure 1C), ELISA (Figure 1D) and Western blotting (Figure 1E) to verify the negative correlation between DHT concentration and IGF‐1 level. The correlation was also verified on prostate primary fibroblasts (Figure S2A,B). The results showed that AR signalling impairment in prostatic stromal cells reduced IGF‐1 secretion, resulting in the abnormal regulation of epithelial cells. Although previous studies have revealed that DHT regulates the secretion of IGF‐1,^15^, ^16^ few studies have focused on how IGF‐1 is associated with autophagy in an androgen‐deficient environment.

### 3.2 5‐ARI suppresses IGF‐1 expression, which has a negative correlation with autophagy in BPH tissues

**Figure 2:**
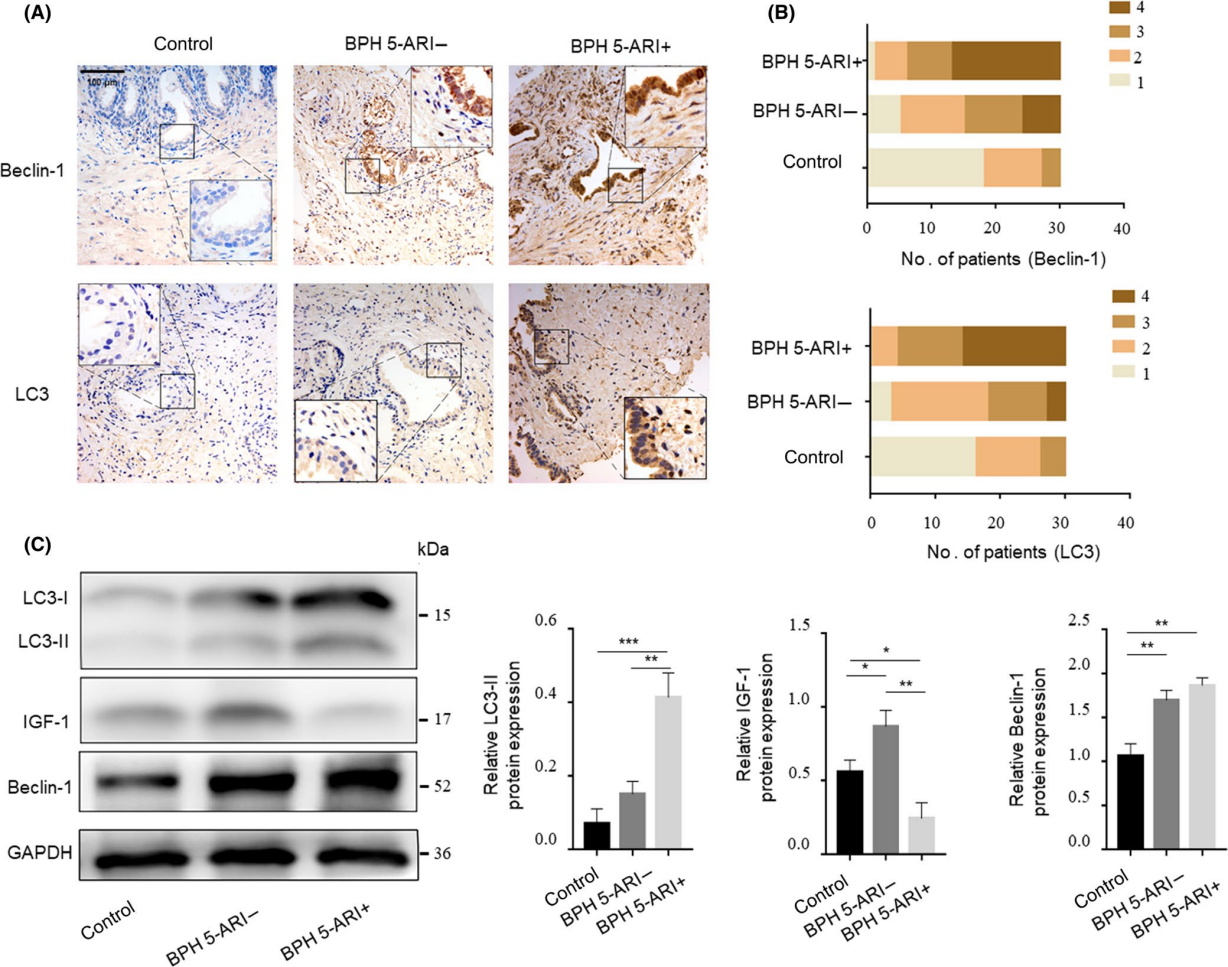
Expression of IGF‐1 and autophagy‐related genes in patient prostate tissues. A, Immunohistochemical analysis of the expression of LC3 and Beclin‐1 in the prostate tissues of the “control,” “BPH 5‐ARI‐” and “BPH 5‐ARI+” groups (scale bars, 100 μm). B, Statistical graph of the IHC results of LC3 and Beclin‐1 expression in the prostate epithelium. (n = 30) (C) Western blot and statistical analysis of LC3‐II, IGF‐1 and Beclin‐1 proteins in different groups. *P < 0.05, **P < 0.01, ***P < 0.001

The effect of IGF‐1 on autophagy in prostate epithelial cells after 5‐ARI treatment and the underlying molecular mechanisms are poorly understood.^13^ To do this, we first evaluate autophagy levels in prostate specimens from patients who received 5‐ARI. The prostate specimens of patients in Shanghai First People's Hospital were divided into 3 groups: control, BPH 5‐ARI‐ and BPH 5‐ARI+. The expression levels of two indicators of autophagy (LC3 and Beclin‐1) in the 3 groups of prostate specimens were examined by IHC (Figure 2A). The results showed that prostate epithelial cells express relatively high levels of LC3 and Beclin‐1 after 5‐ARI treatment in surgical BPH 5‐ARI+ compared with surgical BPH 5‐ARI‐ (Figure 2B, Table S4). We further examined the expression levels of IGF‐1, LC3‐II and Beclin‐1 in those 3 groups by Western blotting. The expression levels of IGF‐1 were higher in Surgical BPH 5‐ARI‐ but lower in Surgical BPH 5‐ARI+, whereas the levels of both LC3‐II and Beclin‐1 were opposite (Figure 2C). These results indicated that the IGF‐1 levels were negatively regulated by 5‐ARI treatment. Additionally, 5‐ARI significantly induced autophagy in the prostate epithelium.

### 3.3 IGF‐1 inhibits autophagic flux through the mTOR pathway in prostate epithelial cells

**Figure 3:**
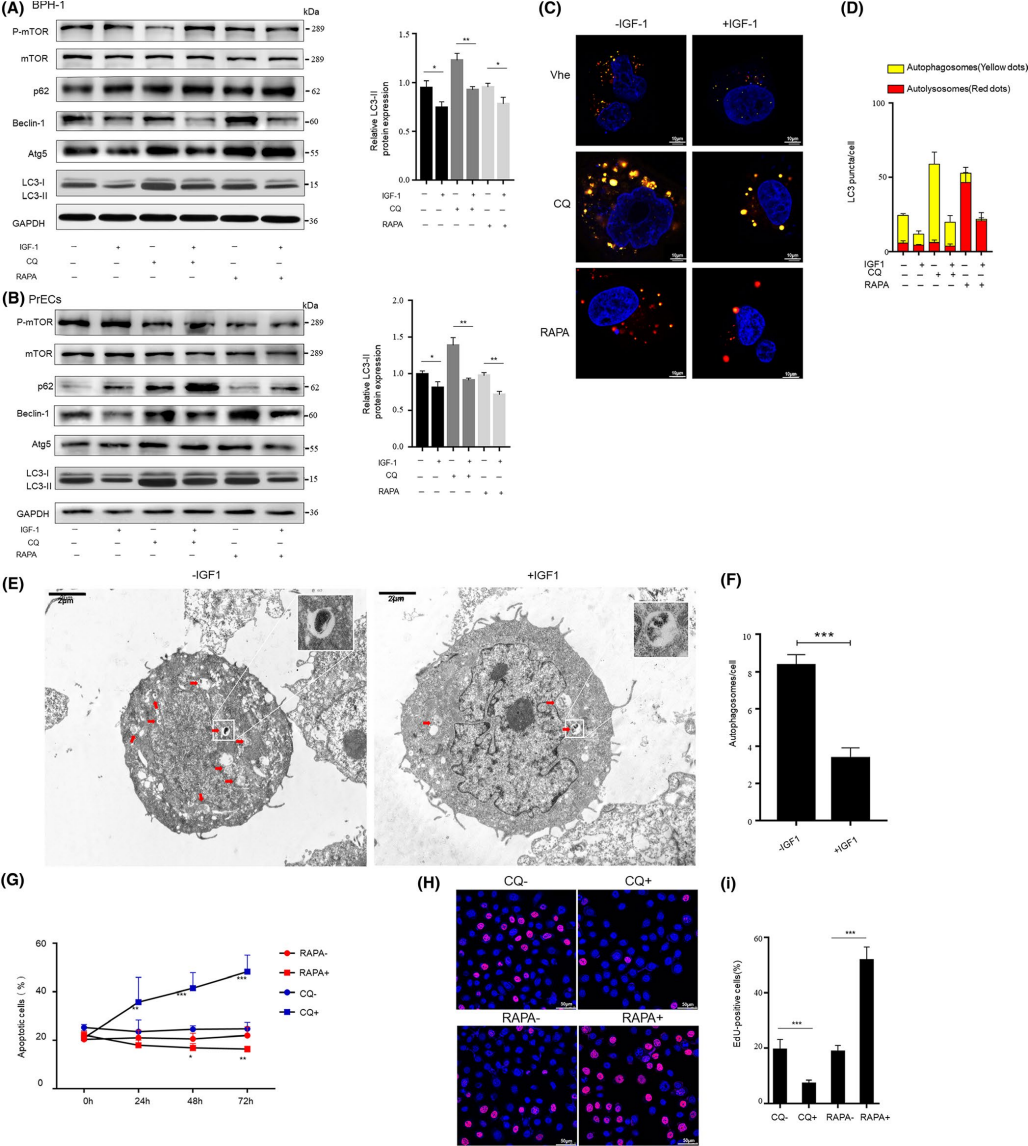
IGF‐1 inhibits autophagy in prostate epithelial cells. A‐D, BPH‐1 or PrECs cells were treated with 100 ng/mL IGF‐1 or ddH_2_O as a vehicle control for 1 h and then with 50 nmol/L RAPA or 50 μmol/L CQ for 1 h. A and B, Left, Western blotting of p‐mTOR, mTOR, p62, Beclin‐1, Atg5 and LC3‐II Right, statistical analysis of relative LC3‐II expression. C & D, The number of autophagosomes and autolysosomes in BPH‐1 transiently overexpressing mRFP‐GFP‐LC3 were observed and analysed under confocal microscope (scale bars: 10 μm). Veh: vehicle control group. E and F, Transmission electron microscope (TEM) observation and statistical analysis of the number of autophagic vacuoles in BPH‐1 treated with 100 ng/mL IGF‐1 or ddH_2_O for 1 h before fixation with 2.5% glutaraldehyde (scale bars: 2 μm). G, Time kinetic curves for the flow cytometry analysis of the apoptotic cell percentage in the BPH‐1 cell population after incubation with 50 nmol/L RAPA, 50 μmol/L CQ or the control treatment for different times. H, Proliferation of BPH‐1 cells after 50 nmol/l RAPA/50 μmol/L CQ/control treatment for 24 h was measured using the EdU kit (scale bars: 50 μm). I, Statistical analysis of the proportion of EdU‐positive cells. RAPA‐ with DMSO as the control group; CQ‐ with ddH2O as the control group. *P < 0.05, **P < 0.01, ***P < 0.001

To determine whether IGF‐1 could induce or inhibit autophagy in prostate epithelial cells, we examined the ultrastructure of prostate epithelial cells treated with IGF‐1 (100ng /mL). At this dose, IGF‐I in the experimental medium activated mTOR phosphorylation. Atg5, Beclin‐1, p62, LC3‐II expression in BPH‐1 and human prostate epithelial cells (PrECs) were also assessed by Western blotting. The results showed that IGF‐1 treatment significantly increased p62 but reduced the expression levels of Beclin‐1 and LC3‐II (Figure 3A,B). Moreover, changes in the expression levels of these proteins after the addition of RAPA and CQ were also monitored. RAPA significantly inhibits the phosphorylation of mTOR and thus affects the expression of other downstream genes; however, CQ inhibits the promotion of autophagic flow. The use of the mRFP‐GFP‐LC3 adenovirus system, which involved fluorescence microscopy, further confirmed the low level of autophagy when cells were cultured with IGF‐1. Since GFP is acid sensitive, GFP fluorescence is quenched when autophagosomes are fused with lysosomes. At the same time, mRFP is used to identify and trace LC3, so only red fluorescence can be detected. The quenching of GFP intuitively and clearly indicated the level of autophagic flow, which was manifested as the fusion of lysosomes and autophagosomes to form autolysosomes. Figure 3C,D show that after being treated with IGF‐1, the numbers of both the yellow LC3 puncta and the red LC3 puncta were markedly lower in BPH‐1 cells regardless of whether autophagic flow was additionally altered by RAPA, CQ or vehicle control. Moreover, the formation and accumulation of autophagosomes or autolysosomes were detected by TEM. The number of autophagic vacuoles was significantly lower in the IGF‐1‐treated BPH‐1 cells (Figure 3E,F). The results of this analysis indicate that the ablation of AR signalling in stromal cells may upregulate autophagy in prostatic epithelial cells via IGF‐1 deficiency. Furthermore, we observed that CQ treatment increased the proportion of apoptotic cells and decreased the number of the EdU‐positive cells among the BPH‐1 cells (Figure 3G,H and I, Figure S3A), which indicated that the blockade of autophagy flux may enhance apoptosis and suppress DNA synthesis in and the cell viability of prostate epithelial cells.

### 3.4 AR signalling impairment in stromal cells stimulates autophagy in prostate epithelial cells

**Figure 4:**
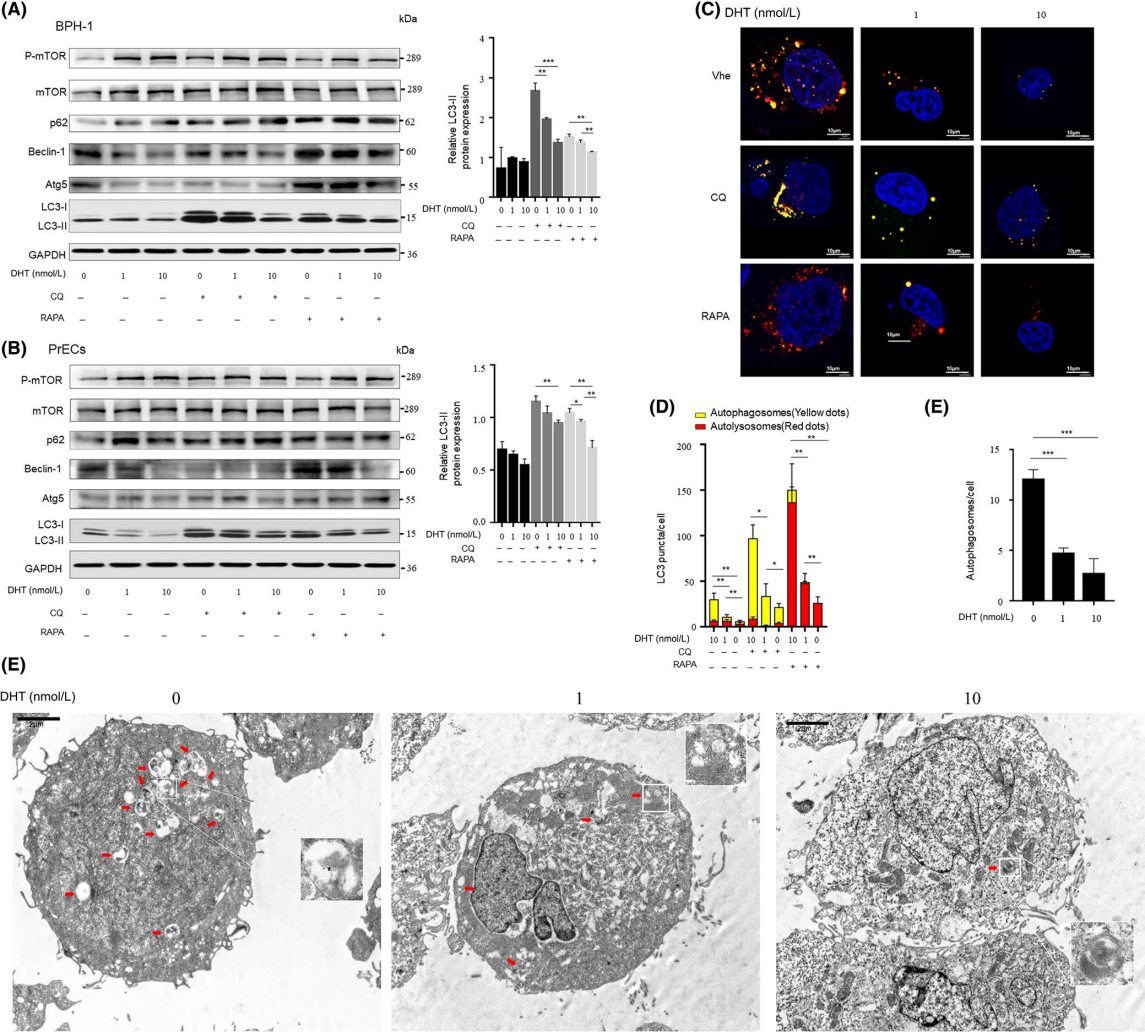
Androgen ablation in prostate fibroblasts promotes autophagic flux in co‐cultured prostate epithelial cells. A and B, BPH‐1 or PrECs cells were treated with conditioned medium from WPMY‐1‐AR cells that were fed for 72 h with 0, 1 or 10 nmol/L DHT, respectively, followed by 50 nmol/L CQ or 50 μmol/L RAPA treatment for another 1 h. Left, Western blotting analysis. Right, statistical analysis of relative LC3‐II expression. C, BPH‐1 cells stably expressing an mRFP‐GFP‐LC3 fusion were treated with conditioned medium for 1 h and then fed with 50 nmol/L RAPA or 50 μmol/L CQ for another 1 h. (scale bars, 10 μm). Veh: vehicle control group. D, Average number of autophagosomes and autolysosomes in BPH‐1 was analysed and plotted. E, TEM results of BPH‐1 cultured with conditioned medium for 1 h before fixation (scale bars: 2 μm) (F) Quantification of the number of autophagic vacuoles. *P < 0.05, **P < 0.01, ***P < 0.001

To verify the hypothesis that AR signalling impairment in prostatic stromal cells stimulated prostatic epithelial autophagy through the down‐regulation of IGF‐1, BPH‐1 cells were incubated with conditioned medium from WPMY‐1‐AR cells or prostate primary fibroblasts that were treated with 0, 1 or 10 nM DHT. BPH‐1 cells were treated with serum‐free, phenol red‐free medium without DHT for 48 hours prior to changing the medium to conditioned medium. The Western blot results showed that as the DHT concentration increased, higher p‐mTOR and p62 expression and lower LC3‐II and Beclin‐1 expression were induced by the conditioned medium in BPH‐1 and PrECs (Figure 4A,B, Figure S2C). Low DHT‐induced autophagy was enhanced by RAPA, indicating that the androgen/AR signalling blockade regulated the secretion of cytokines by prostate stromal fibroblasts via the mTOR‐dependent pathway and thereby promoted the formation of autophagic flux in prostate epithelial cells. Moreover, mRFP‐GFP‐LC3‐expressing BPH‐1 cells that were cultured in conditioned medium and extracted with low‐concentration‐DHT‐treated WPMY‐1‐AR cells showed increased numbers of autophagosomes (mRFP+/GFP+ puncta) and autolysosomes (mRFP+ puncta) (Figure 4C,D). Additionally, the formation of autophagosomes was detected by TEM in BPH‐1 cells incubated with low‐concentration‐DHT‐treated conditioned medium (Figure 4E,F, Figure S2D,E). These data revealed that prostate fibroblasts with impaired AR signalling promote autophagy in co‐cultured prostate epithelial cells.

### 3.5 Autophagy in prostate epithelial cells regulated by fibroblasts is reduced by IGF‐1 receptor inhibitors

**Figure 5:**
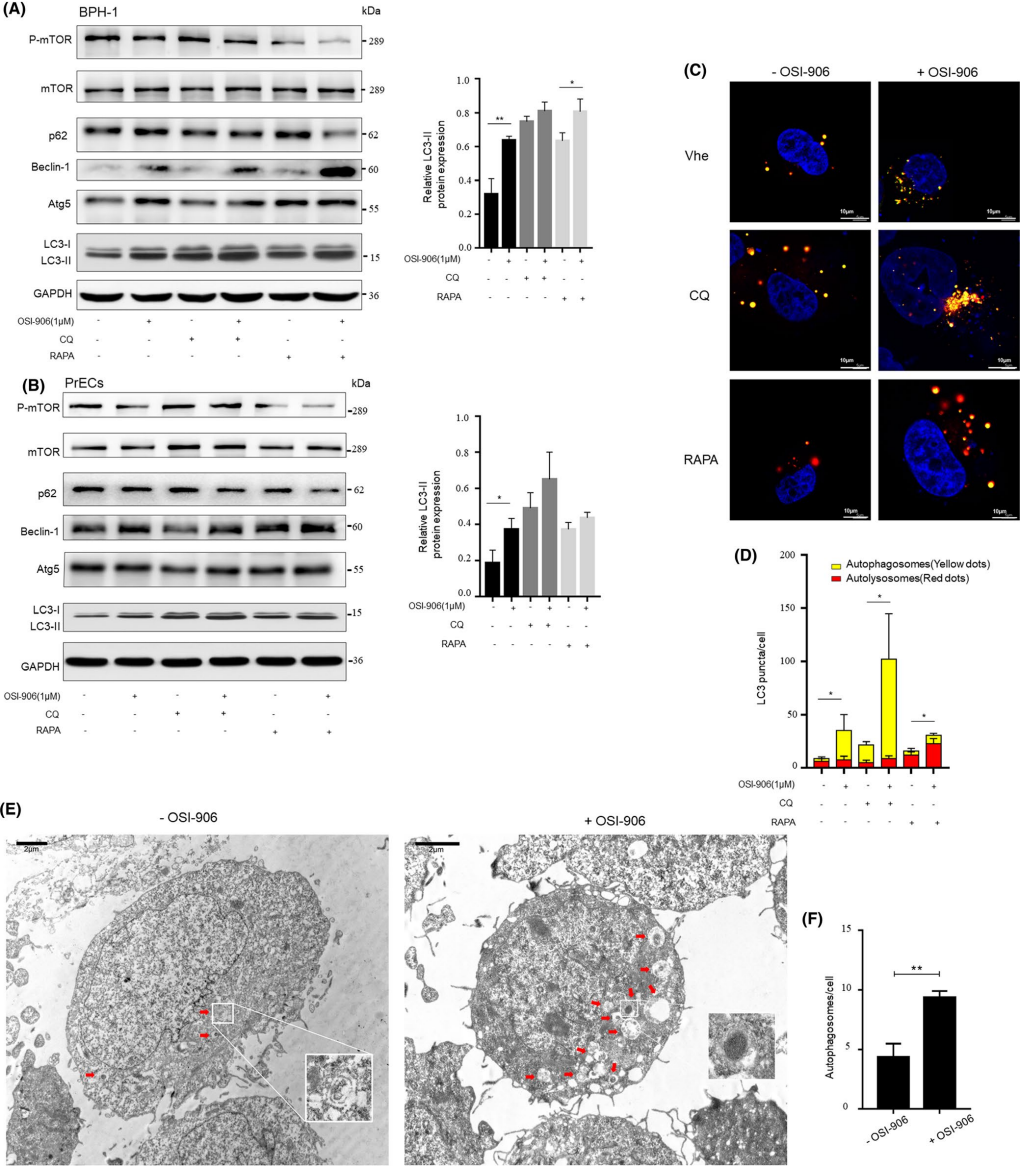
Assessment of autophagy in prostate epithelial cells regulated non‐cell autonomously by fibroblasts after treatment with the IGF‐1 receptor inhibitor OSI‐906. A & B, BPH‐1 or PrECs pre‐treated with the IGF‐1 receptor antagonist OSI‐906 (1 mol/L) or DMSO as the vehicle control for 1 h were incubated for 1 h with WPMY‐1‐AR conditioned medium from cells that were cultured 72 h with 10 nmol/L DHT, followed by 50 nmol/L RAPA or 50 μmol/L CQ treatment for another 1 h. Left, Western blotting. Right, statistical analysis of relative LC3‐II expression. C, Confocal microscopy of BPH‐1 cells transfected with mRFP‐GFP‐LC3 adenovirus and incubated for 1 h with 10 nmol/L DHT conditioned medium with pre‐treatment of OSI‐906 (1 mol/L) or DMSO to assess autophagic flux. (scale bars: 10 μm). Veh: vehicle control group. D, Quantification of the average number of autophagosomes and autolysosomes in BPH‐1. E, TEM to detect autophagic vacuoles in BPH‐1 cultured with conditioned medium for 1 h with OSI‐906 (1 mol/L) or DMSO pre‐treatment before fixation. (scale bars: 2 μm) (F) The number of autophagic vacuoles in BPH‐1 was quantitated. *P < 0.05, **P < 0.01, ***P < 0.001

After BPH‐1 cells were starved with serum‐free medium, the cells were pre‐treated with IGF‐1 receptor antagonist OSI‐906 (1 mol/L) or DMSO as the vehicle control for 1 hour and treated for another hour with conditioned medium extracted from WPMY‐1‐AR cells that were treated with 10 nmol/L DHT. The levels of Beclin‐1, LC3‐II were higher, and the levels of p‐mTOR and p62 were lower in BPH‐1 cells and PrECs that were pre‐treated with OSI‐906 than in cells treated with the vehicle control (Figure 5A,B). Next, the tendency of OSI‐906 to promote autophagic flux, which was down‐regulated by conditioned medium at high concentrations of DHT, was further confirmed by observing whether the formation rate of yellow LC3 puncta and red LC3 puncta increased when BPH‐1 cells were pre‐treated with OSI‐906 (Figure 5C,D). Consistent with the Western blotting and mRFP‐GFP‐LC3 adenovirus system results, the TEM experiments revealed more autophagosomes and autolysosomes in BPH‐1 cells treated with OSI‐906 (Figure 5E,F). The results indicate that IGF‐1 receptor antagonist OSI‐906 promotes the down‐regulation of autophagic flux in prostate epithelial cells that are non‐cell autonomously regulated by stromal cells without AR signalling impairment.

### 3.6 Chloroquine diphosphate inhibits autophagy and hinders the formation of recombinant grafts in vivo

**Figure 6:**
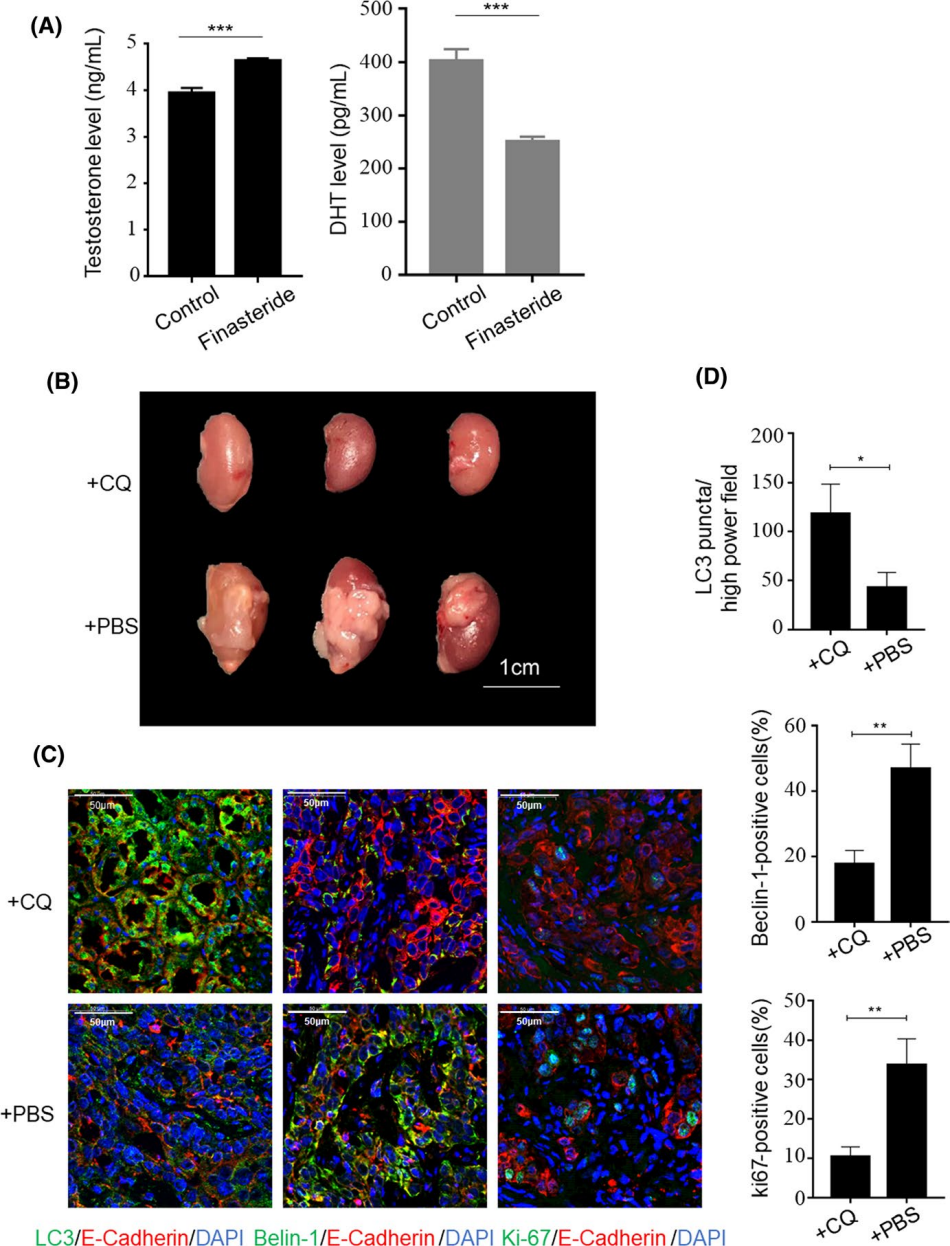
The effect of chloroquine diphosphate on recombinant grafts in vivo*. *A, Elisa analysis of testosterone and DHT in *nude* mice serum treated with finasteride for one week. An equal volume of DMSO was used as a control. B, Image of recombinant grafts formed by a mixture of WPMY‐1‐AR and BPH‐1 cells in nude mice. Scale bars, 1 cm. +CQ: chloroquine diphosphate group; +PBS: PBS treatment as the control group. C, Immunofluorescence staining of autophagy and proliferation markers. LC3 (green) was used to verify that the degradation of the autophagosome‐encapsulated contents was blocked by chloroquine. Beclin‐1 (green), autophagy marker; Ki‐67 (green), the proliferation marker. E‐cadherin (red) was used as an epithelium marker to define compartment of recombinant grafts (scale bars, 50 μm). D, Statistical analysis of the number of LC3 puncta, Beclin‐1‐positive and Ki‐67‐positive prostate epithelial cells. *P < 0.05, **P < 0.01

To explore the viability of BPH‐1 cells co‐cultured with WPMY‐1‐AR in vivo, grafts consisting of a mixture of WPMY‐1‐AR and BPH‐1 cells were implanted under the renal capsule of each male nude mouse. Nude mice were subcutaneously treated with finasteride for androgen ablation to simulate BPH patients receiving long‐term 5‐ARI treatment. The level of serum testosterone and DHT of nude mice after one‐week finasteride treatment was shown in Figure 6A. After 12 weeks, the kidneys of the nude mouse were excised, and then, all of the renal capsules under which the graft was seeded was peeled off and fixed in formalin. Regarding the appearances of the grafts, the volume of the grafts in the CQ‐treated group (intraperitoneally injected with CQ, 50 mg/kg per day) was significantly smaller than that of the vehicle control group (intraperitoneally injected with phosphate buffered saline (PBS)) (Figure 6B). CQ inhibited the promotion of autophagic flux by disrupting the acidic environment in the lysosomes and blocking the degradation of the autophagosome‐encapsulated contents, including LC3.^17^ As shown in Figure 6C,D, in the CQ‐treated group, LC3 formed more puncta and was scattered in the cytoplasm. Therefore, this phenomenon suggested that CQ inhibited the process of autophagosome‐lysosome fusion. In addition, the CQ‐treated group showed reduced Beclin‐1 and Ki‐67 in E‐cadherin‐positive cells than the vehicle control group. The data indicate that CQ down‐regulates the level of autophagy and inhibits the proliferation of prostate epithelial cells in vivo.

## 4 DISCUSSION

Insulin‐like growth factor‐1 (IGF‐1) is one of the insulin‐like proteins that can affect cell differentiation, proliferation, apoptosis, and organismal growth and development.^18^ Epidemiological studies have suggested that elevated levels of IGF‐1 are associated with an increased risk of BPH.^19^ IGF‐1 is also a key factor that mediates the transduction of prostate stromal‐epithelial cell signalling. Li *et al* 20 found that prostate epithelial cells are regulated by IGF‐1 released by stromal cells in a non‐cell‐autonomous manner. Additionally, IGF‐1 can cooperate with DHT and basic fibroblast growth factor to promote prostate epithelial cell proliferation. Thus, most previous studies have indicated that IGF‐1 has growth stimulatory and anti‐apoptotic effects on prostate tissue.^21^, ^22^ The effect that IGF‐1 promotes prostatic epithelial cells proliferation was also investigated in our study (Figure S3B,C). However, the inhibitory effect of IGF‐1 on autophagy has been reported in human fibroblasts,^23^ mammary epithelial cells^24^ and human osteocarcinoma cells^25^ via the mTOR pathway.

Here, we demonstrated that the long‐term use of 5‐ARIs can reduce IGF‐1 expression in the epithelial cells of the prostate transitional zone. Conversely, the autophagy level is significantly increased. In addition, we found that after DHT stimulation, the levels of IGF‐1 secreted by prostate stromal cells also decrease. Decreased IGF‐1 significantly induces autophagy, promoting the proliferation of prostate epithelial cells and increasing the graft volume in vivo. BPH progression that is triggered by the promotion of autophagic flux depends on (a) the inhibition of IGF‐1 release, (b) high levels of autophagy, and (c) the upregulation of proliferation and down‐regulation of apoptosis.

First, DHT regulates IGF‐I expression in prostatic stromal cells in a dose‐ and time‐dependent manner, thereby modulating prostatic epithelial cell mitosis and apoptosis.^16^ Combined with our IHC and in vitro results, which show the ability of 5‐ARI treatment to suppress the levels of IGF‐1 in prostate tissues, our findings indicate that AR signalling impairment restrains the release of IGF‐1 from prostate stromal cells. Second, IGF‐1 deficiency induces autophagy in prostatic epithelial cells. IGF‐1 that is secreted by stromal cells regulates epithelial autophagy via the mTOR pathway. The underlying mechanism is as follows: IGF‐1 activates AKT phosphorylation at multiple sites on TSC2 to inhibit the GTPase activity of TSC2, leading to the activation of mTORC1.^26^ In addition, PI3K activation in the IGF‐1/AKT pathway also upregulates mTORC1, thereby inhibiting the downstream autophagy signalling pathway.^27^ Unfortunately, whether IGF‐1 regulates BPH via the autophagy pathway remains unclear. Our previous study mainly focused on the relationship between autophagy and prostate stromal cells because the stromal compartment accounts for over 60% of BPH tissue.^28^ Here, our observations are consistent with previous findings that androgen deprivation induces autophagy in prostatic fibroblasts (Figure S4). Nevertheless, autophagy‐related genes were highly expressed in the prostate epithelium rather than in the stroma.^29^ Therefore, an investigation of the role of autophagy in prostate epithelial cells would be more meaningful. A previous study reported that androgen suppression elevated the autophagy level in benign prostatic epithelial cells.^13^ We hypothesize that with the long‐term use of 5‐ARIs, the level of autophagy in prostate epithelial cells will be increased due to the weakened capacity of stromal cells to secrete IGF‐1. However, because AR is poorly expressed in BPH‐1 and PrECs, the direct effects of androgens on prostate epithelial cells can be ruled out in vitro.^30^, ^31^ The distributions of AR in prostate stromal cells and epithelial cells and the sensitivity of IGF‐1 receptor in epithelial cells are different for each individual. Hence, an assessment of these congenital variables may help to formulate a therapeutic strategy that addresses the different mechanisms of BPH progression.

Third, the activation of autophagy can promote prostatic epithelial cell proliferation and reduce apoptosis. We observed that prostate epithelial cell autophagy is an important factor in maintaining proliferation and inhibiting apoptosis. Furthermore, the concept of the "autophagy/apoptosis checkpoint," which allows cells to reach different endings, namely survival and death, has been established.^32^ Specifically, senescent prostate epithelial cells will reach an ultimate tipping point, and if intracellular autophagic pathways in these cells are activated at this point, these cells will be shifted away from death. Together with the results of the present study showing that prostatic epithelial cell proliferation is regulated by the formation of autophagic flux, these findings demonstrate the crucial role of crosstalk between autophagy, apoptosis and proliferation pathways in prostatic pathogenesis. Collectively, our study revealed the mechanism underlying how the 5‐ARI treatment effects are influenced by the relationship between autophagy and the non‐cell‐autonomous regulation of prostate epithelial cells, further confirming the potential utility of autophagic inhibitory approaches to prevent BPH progression.

## CONFLICT OF INTEREST

The authors declare that they have no conflict of interest in relation to this work.
