# Supplementary figures and images for: 5‐ARI induces autophagy of prostate epithelial cells through suppressing IGF‐1 expression in prostate fibroblasts
Source: Cell Prolif. 2019 Mar 18;52(3):e12590. doi: 10.1111/cpr.12590 (PMC6536403; doi:10.1111/cpr.12590)

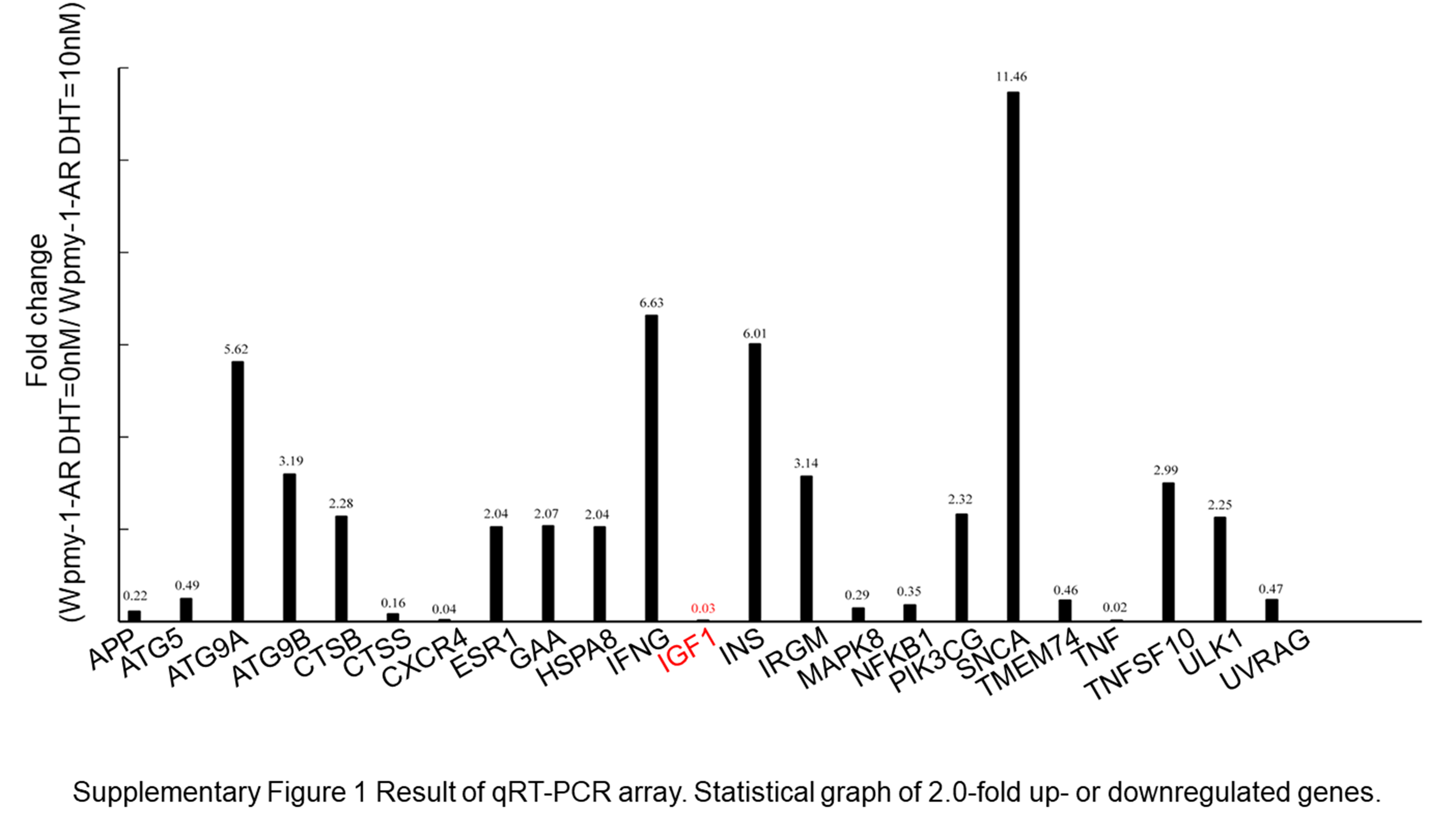

Supplement: Supplementary file 1 [file CPR-52-e12590-s001.tif]

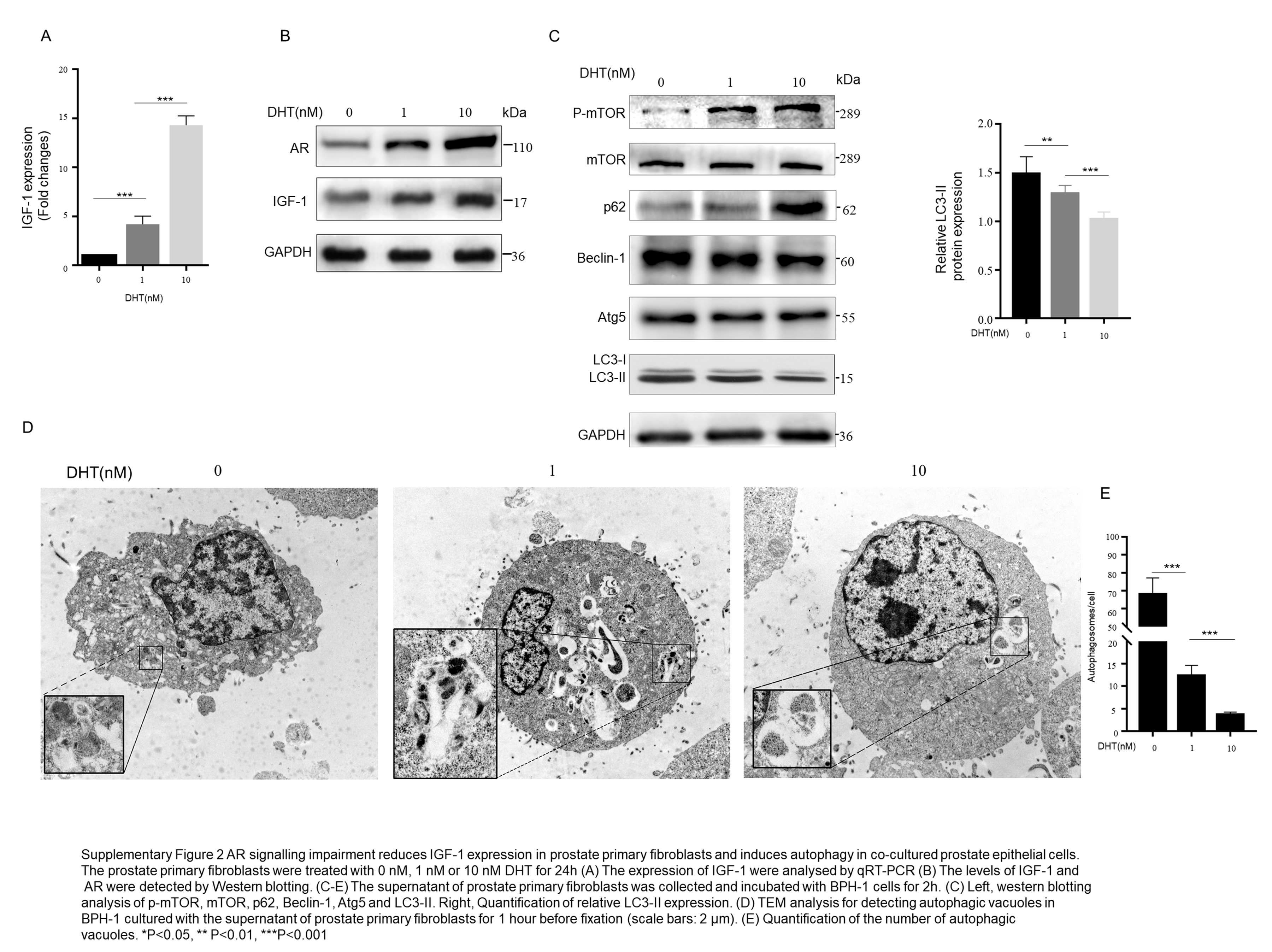

Supplement: Supplementary file 2 [file CPR-52-e12590-s002.tif]

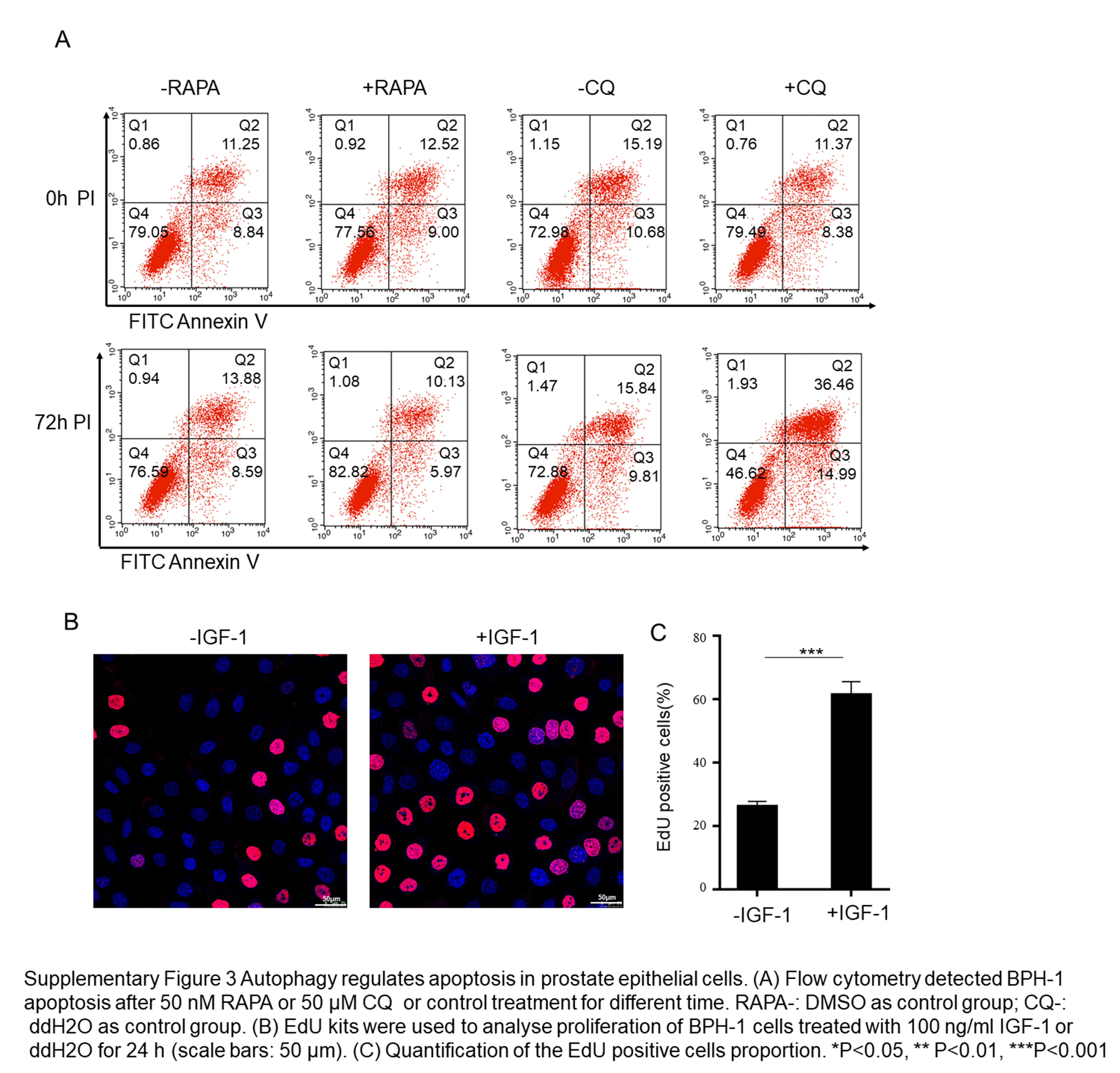

Supplement: Supplementary file 3 [file CPR-52-e12590-s003.tif]

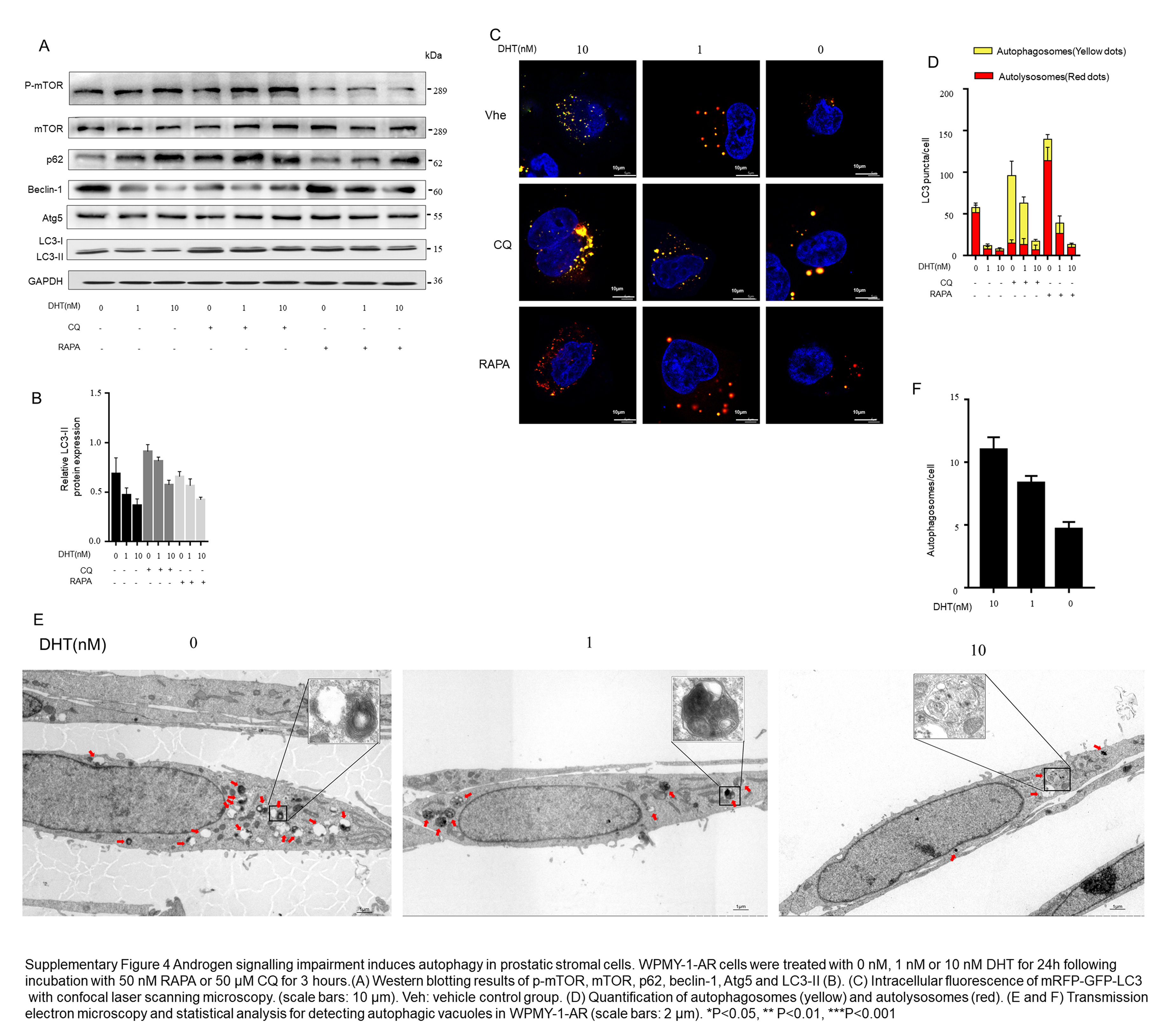

Supplement: Supplementary file 4 [file CPR-52-e12590-s004.tif]
