# Supplementary material for: 5‐ARI induces autophagy of prostate epithelial cells through suppressing IGF‐1 expression in prostate fibroblasts
Source: Cell Prolif. 2019 Mar 18;52(3):e12590. doi: 10.1111/cpr.12590 (PMC6536403; doi:10.1111/cpr.12590)
Supplement: Supplementary file 6 [file CPR-52-e12590-s006.docx]

**Supplementary Table 2** BPH, benign prostatic hyperplasia; 5-ARI -, patients weren't treated with 5α-reductase inhibitor for at least six months before surgery; 5-ARI +, patients treated once daily with 5α-reductase inhibitor for at least six months before surgery（PV: prostate volume; Qmax: maximum urinary flow rate; PVR: postvoid residual; IPSS: international prostate symptom score; PSA: prostate specific antigen; QoL: quality of life; NA:not available）.

| Preoperative | BPH 5-ARI - (n=30) | BPH 5-ARI +(n=30) | P value |
| --- | --- | --- | --- |
| Parameters | mean(range) | mean(range) |  |
| Age (year) | 63.7（56-82） | 65.2（58-79） | 0.397 |
| PV (ml) | 60.4（38.9-88.4） | 52.5（40.8-73.3） | 0.023 |
| Qmax (ml/s) | 6.8（5.3-10.8） | 7.2（5.9-11.6） | 0.476 |
| PVR (ml) | 85.5（44.9-160.3） | 80.5（38.8-110.5） | 0.368 |
| IPSS | 21.5（16-28） | 20.2（15-27） | 0.569 |
| PSA（ng/ml） | 4.2（1.3-7.2） | 3.3（2.1-5.7） | 0.064 |
| QoL | 4.4（3-6） | 4.2（3-6） | 0.668 |
