# Supplementary material for: 5‐ARI induces autophagy of prostate epithelial cells through suppressing IGF‐1 expression in prostate fibroblasts
Source: Cell Prolif. 2019 Mar 18;52(3):e12590. doi: 10.1111/cpr.12590 (PMC6536403; doi:10.1111/cpr.12590)
Supplement: Supplementary file 7 [file CPR-52-e12590-s007.docx]

**Supplementary Table 3** Contingency table showing number of patients in each group and IHC score stratification. IHC score was defined according to IGF1、SNCA、TNF-α、CXCR4 or IFNG staining intensity in the stromal compartment of the prostate tissue.

| IHC score | BPH 5-ARI – (n=30) | BPH 5-ARI + (n=30) |
| --- | --- | --- |
| Stromal IGF-1 expression |  |  |
| 1 | 1 (3.3 %) | 11 (36.7 %) |
| 2 | 5 (16.7 %) | 15 (50.0 %) |
| 3 | 8 (26.7 %) | 3 (10.0 %) |
| 4 | 16 (53.3 %) | 1 (3.3 %) |
| Stromal SNCA expression |  |  |
| 1 | 11 (36.7 %) | 11 (36.7 %) |
| 2 | 17 (56.7%) | 15 (50.0 %) |
| 3 | 2 (6.7 %) | 4 (13.3 %) |
| 4 | 0 (0 %) | 0 (0 %) |
| Stromal TNF-α expression |  |  |
| 1 | 17 (56.7%) | 16 (53.3 %) |
| 2 | 10 (33.3 %) | 11 (36.7 %) |
| 3 | 3 (10.0 %) | 3 (10.0 %) |
| 4 | 0 (0 %) | 0 (0 %) |
| Stromal CXCR4 expression |  |  |
| 1 | 17 (56.7%) | 18 (60.0 %) |
| 2 | 9(30.0 %) | 10 (33.3 %) |
| 3 | 4 (13.3 %) | 2 (6.7 %) |
| 4 | 0 (0 %) | 0 (0 %) |
| Stromal IFNG expression |  |  |
| 1 | 2 (6.7 %) | 6 (20.0%) |
| 2 | 15 (50.0 %) | 11 (36.7 %) |
| 3 | 9(30.0 %) | 10 (33.3 %) |
| 4 | 4 (13.3 %) | 3 (10.0 %) |
| IHC, immunohistochemistry; BPH, benign prostatic hyperplasia; 5-ARI -, without 5α-reductase inhibitor treatment; 5-ARI +, with 5α-reductase inhibitor treatment. | | |
