# Supplementary material for: 5‐ARI induces autophagy of prostate epithelial cells through suppressing IGF‐1 expression in prostate fibroblasts
Source: Cell Prolif. 2019 Mar 18;52(3):e12590. doi: 10.1111/cpr.12590 (PMC6536403; doi:10.1111/cpr.12590)
Supplement: Supplementary file 8 [file CPR-52-e12590-s008.docx]

**Supplementary Table 4** Contingency table showing number of patients in each group and IHC score stratification. IHC score was defined according to LC3 or Beclin-1 staining intensity in the stromal compartment of the prostate tissue.

| IHC score | control (n=30) | BPH 5-ARI – (n=30) | | BPH 5-ARI + (n=30) |  |
| --- | --- | --- | --- | --- | --- |
| Stromal LC3 expression |  | |  |  |  |
| 1 | 16 (53.3 %) | | 3 (10.0 %) | 0 (0.0%) |  |
| 2 | 10 (33.3 %) | | 15 (50.0%) | 4 (13.3 %) |  |
| 3 | 4 (13.3 %) | | 9 (30.0 %) | 10 (33.3 %) |  |
| 4 | 0 (0.0%) | | 3 (10.0 %) | 16 (53.3 %) |  |
| Stromal Beclin-1 expression |  | |  |  |  |
| 1 | 18 (60.0 %) | | 5 (16.7 %) | 1 (3.3 %) |  |
| 2 | 9 (30.0 %) | | 10 (33.3 %) | 5 (16.7%) |  |
| 3 | 3 (10.0 %) | | 9(30.0 %) | 7 (23.3 %) |  |
| 4 | 0 (0.0 %) | | 6 (20.0 %) | 17 (56.7%) |  |
| IHC, immunohistochemistry; BPH, benign prostatic hyperplasia; 5-ARI -, without 5α-reductase inhibitor treatment; 5-ARI +, with 5α-reductase inhibitor treatment. | | | | | |
