# Supplementary material for: 5‐ARI induces autophagy of prostate epithelial cells through suppressing IGF‐1 expression in prostate fibroblasts
Source: Cell Prolif. 2019 Mar 18;52(3):e12590. doi: 10.1111/cpr.12590 (PMC6536403; doi:10.1111/cpr.12590)
Supplement: Supplementary file 9 [file CPR-52-e12590-s009.docx]

**1.Materials and Methods**

**1.1 Prostate samples**

The fresh transition zones of prostate specimens were collected from consenting patients receiving thulium laser resection of the prostate (TmLRP) at Shanghai General Hospital (Shanghai, China) for symptomatic BPH with LUTS. These patients were further divided into three groups, referred to here as “control”, “BPH 5-ARI-” and “BPH 5-ARI+”, based on whether they had been medicated with 5 mg finasteride once daily for at least six months before surgery. The samples in “control” group were obtained from patients who underwent radical prostatectomy and under 70 years of age. All specimens were pathologically evaluated to exclude tumor tissue. Additionally, specimens in the control group were pathologically verified to exclude hyperplastic tissue. Detailed preoperative basic information of the patients is shown in Supplementary Table 4. For the experiments involving human subjects, all procedures were approved by the Shanghai First People’s Hospital Medical Ethics Committee. Written informed consent was obtained from each patient before study enrolment.

**1.2 Cell culture**

PrECs and prostate primary fibroblasts from prostate samples were isolated and primarily cultured as described previously^1^. The immortalized human prostate epithelial BPH-1 cell line was developed in our laboratory and maintained in RPMI 1640 medium (HyClone, South Logan, UT, USA). The immortalized prostate stromal WPMY-1 cell line was purchased from the American Type Culture Collection (ATCC) (Manassas, VA, USA) and cultured in Dulbecco’s Minimal Essential Medium (DMEM) (HyClone). To explore the role of AR signalling in prostate stromal fibroblasts, we constructed WPMY-1-AR cells in which the AR gene was stably expressed, as described previously^2^. All cells were cultured in medium supplemented with 10% foetal bovine serum (FBS) (Gibco, Grand Island, NY, USA) and 1% penicillin-streptomycin (Gibco) at 37°C with 5% CO2. Before IGF-1 or DHT treatment, cells were starved via culture in phenol red-free DMEM (Gibco) without FBS for 48 hours. Conditioned medium from stromal cells was harvested after 72 hours of culture with different concentrations of DHT in fresh, serum-free, phenol red-free medium. BPH-1 and BPEs were incubated in conditioned medium for 1 h.

**1.3 Antibodies and reagents**

LC3 (#12741), Atg5 (#12994), SQSTM1/p62 (#23214), mTOR (#2983), Phospho-mTOR (#5536) and HRP-linked anti-rabbit IgG (#7074) antibodies were purchased from Cell Signaling Technology (Danvers, MA, USA). Beclin-1 (ab62557), IGF1 (ab9572), Ki-67 (ab15580) and E-cadherin (ab1416) antibodies were purchased from Abcam (Cambridge, UK). GAPDH (D110016) antibody was purchased from Sangon Biotech (Shanghai, China). Alexa Fluor 488-conjugated goat anti-rabbit IgG antibody and rhodamine-conjugated goat anti-mouse IgG antibody were purchased from Jackson ImmunoResearch (West Grove, PA, USA). DHT was obtained from Sigma (St Louis, MO, USA) and used at 1 nM or 10 nM. DHT was dissolved in ethanol. Rapamycin (RAPA, HY-10219) and chloroquine diphosphate (CQ, HY-17589) were procured from MCE (Monmouth Junction, NJ, USA). RAPA was dissolved in dimethylsulfoxide (DMSO) (Sigma), and CQ was dissolved in ultrapure H2O. Finasteride (S1197) was obtained from Selleck (Shanghai, China) and dissolved in DMSO. OSI-906 (Linsitinib) was purchased from Selleck (Shanghai, China) and used at 1 μM. OSI-906 was diluted in DMSO.

**1.4 mRFP-GFP-LC3 adenovirus infection**

mRFP-GFP-LC3 adenovirus was obtained from Genomeditech (Shanghai, China) to track autophagosome maturation. Cells were seeded on 24-well plates and reached approximately 60% confluence at the time of infection. For adenovirus infection, cells were infected using polybrene (Obio, Shanghai, China) according to the manufacturer’s instructions. After infection for 48 h, cells were treated with different reagents and then with 50 nM RAPA or 50 μM CQ for 1 h. Autophagy was observed under a Leica TCS SP8 confocal microscope (Wetzlar, Germany). Autophagic flux was determined by counting the number of yellow puncta (autophagosomes) and red puncta (autolysosomes). More than 10 cells from each group were analysed. Images were acquired with a Leica TCS SP8 confocal microscope.

**1.5 Apoptosis and proliferation assays**

The detection of apoptotic cells was performed by flow cytometry using the Annexin V-FITC Apoptosis Detection Kit (BD Biosciences, San Diego, CA, USA) in accordance with the manufacturer’s instructions. BPH-1 cells were incubated overnight in six-well plates at a density of 5×105 cells per well. After 48 h of FBS starvation, cells were exposed to 50 nM RAPA or 50 μM CQ for different times. Then, cells were collected with 0.25% trypsin (Gibco) and washed twice with ice-cold PBS (HyClone). After resuspension and staining with Annexin V-FITC and PI, cell fluorescence was immediately detected with a BD Accuri C6 flow cytometer (BD Biosciences). DNA synthesis was measured using the EdU KIT (RIBOBIO, Guangzhou, China) according to the manufacturer’s protocols. Cells were directly screened for IGF using a Leica TCS SP8 confocal microscope.

**1.6 Transmission electron microscopy (TEM)**

For analysing autolysosome formation, cells were seeded in six-well plates at a density of 5×10^5^ cells per well and treated as described above. Cells were then collected, fixed in 2.5% glutaraldehyde for 2 h, incubated for another 20 min with 1% OsO_4_ buffer, washed and dehydrated with a series of increasing concentrations of ethanol solutions. Subsequently, cells were embedded in epoxy resin. Next, samples were cut into 70-90-nm-thick ultrathin sections and examined using a CM-120 electron microscope (Philip, Eindhoven, Netherlands).

**1.7 Quantitative real-time polymerase chain reaction (qRT-PCR) and qRT-PCR array**

Total RNA was extracted from prostate samples or cells using the TRIzol reagent (Life Technologies, Foster City, CA, USA). cDNA synthesis was conducted using the PrimeScript RT reagent kit 127 (Takara, Otsu, Shiga, Japan) according to the manufacturer’s guidelines. The reactions for the qRT-PCR analysis were carried out in triplicate for each sample using SYBR Premix Ex Taq (Takara). GAPDH was used as the internal control. Relative mRNA expression was calculated on the basis of the 2-ΔΔCt method. The primers used in this study were as follows: GAPDH forward 5’-GCTCTTCAGTTCGTGTGTGGA-3’, reverse 5’-GCCTCCTTAGATCACAGCTCC-3’ and IGF-1 forward 5’-GGAGCGAGATCCCTCCAAAAT-3’, reverse 5’-GGCTGTTGTCATACTTCTCATGG-3’. The expression levels of autophagy-associated genes were screened using the RT² Profiler™ PCR Array (PAHS-084Z, Qiagen, Hilden, Germany) as described previously^2^.

**1.8 Western blotting**

Harvested cells or collected tissues for Western blotting were lysed in RIPA reagent (Beyotime, Suzhou, China) containing a phosphatase inhibitor (if necessary) and protease inhibitor (Thermo, Wilmington, DE, USA). Proteins were quantified using a Pierce BCA Protein Assay kit (Thermo Scientific, Waltham, MA, USA) and separated on 7.5-15% SDS-polyacrylamide gels. Next, proteins were transferred to polyvinylidene fluoride (PVDF) membranes (Millipore, Billerica, MA, USA). Membranes were blocked with 10% bovine serum albumin (BSA) or 5% non-fat dry milk in Tris-buffered saline Tween 20 (TBST) for 90 min at room temperature and incubated overnight with appropriate dilutions of the primary antibodies described above, followed by incubations with anti-rabbit horseradish peroxidase antibody for 90 min. Immunoreactive bands were detected using the ChemiLucent ECL Detection system (Millipore), and sensitometry analysis was performed with the ImageJ programme (National Institutes of Health).

**1.9 Enzyme-linked immunosorbent assay (ELISA)**

IGF-1 levels were analysed in the cell culture supernatants collected from experiments terminated at 48 h. The serum of *nude* mice was collected for ELISA experiments. IGF-1 ELISA kits were commercially available from eBioscience. [Testosterone](http://www.baidu.com/link?url=dY6RZnx5PYAvkm4e5WYRspvsN8mgn0DSjGEMi02FwGPUmMP6waDFDQqG5OUcW4Zgu6nJbSBFkpDaaEOIA6CeUyaEJowUU9S5GDfMq0gpd5_) and DHT ELISA kits were purchased form CUSABIO (Wuhan, China).The procedures were performed according to the manufacturer’s instructions. The optical density (OD) was measured with a plate reader.

**1.10 *In vivo* cell recombination**

Six- to eight-week-old male *nude* mice were supplied by the Animal Center of the Chinese Academy of Sciences. The *nude* mice had been preventatively treated subcutaneously with finasteride (20 mg/kg per day) starting one week before graft implantation. A total of 2×10^5^ WPMY-1-AR and BPH-1 cells at a ratio of 1:1 were resuspended in Matrigel (Corning, New York, NY, USA) to a volume of 50 μl per graft for each male *nude* mouse, and the mixture was then injected under the renal capsule. After implantation, *nude* mice in each group were further subjected to intraperitoneal (i.p.) injection with either CQ (50 mg/kg per day) or an equal volume of PBS for 11 weeks. On the twelfth week, all mice were killed by isoflurane inhalation, and the grafts under the renal capsules were collected for immunofluorescence. Animal experiments were approved and performed in accordance with protocols of the Medical Science Ethics Committee at Shanghai First People’s Hospital of Shanghai Jiao Tong University.

**1.11 Immunohistochemistry (IHC)**

To determine the expression levels of IGF-1, Beclin-1, and LC-3, immunohistochemistry (IHC) staining was carried out on 5-μm sections of paraffin-embedded prostate specimens that were fixed in 4% formalin. Briefly, the slides were treated with the primary antibody overnight followed by secondary antibody for 1 h according to the manufacturer’s instructions. Additionally, another section on each slide was incubated with PBS as a negative control. The proportion of positively stained cells was scored semi-quantitatively as follows: negative stain­ing (1); weakly positive staining (2); moderately positive stain­ing (3); and strongly positive staining (4). IHC images were captured with a Leica DM5500 B microscope.

**1.12 Immunofluorescence (IF)**

Three-micron-thick sections were acquired from frozen blocks of graft specimens and mounted on Super Frost Plus slides. Sections were fixed in acetone for 15 min followed by rinsing with PBS-Tween 20 (PBST). Next, slides were blocked with 1% BSA in PBS and then treated with primary antibodies according to the manufacturer’s instructions. After washing in PBST, slides were treated with 1% BSA containing secondary antibodies with fluorescent conjugates. Lastly, the sections were washed in PBST and incubated with Gold Antifade Mountant containing DAPI (Thermo) and stored in the dark at 4°C. Immunofluorescence images were captured on a Leica DMi8 fluorescence microscope.

**1.13 Statistical analyses**

All statistical analyses were conducted using SPSS (version 19.0.0, SPSS Inc., Chicago, IL). We expressed the data as the mean ± standard deviation (SD). We determined statistical significance by two-sided Student’s t-test or one-way ANOVA. P < 0.05 was considered statistically significant. All experiments were repeated three times.

**References**

1. Zhao FJ, Han BM, Yu SQ, Xia SJ. Tumor formation of prostate cancer cells influenced by stromal cells from the transitional or peripheral zones of the normal prostate. *Asian J Androl.* 2009;11(2):176-182.
2. Jiang CY, Yang BY, Zhao S, et al. Deregulation of ATG9A by impaired AR signaling induces autophagy in prostate stromal fibroblasts and promotes BPH progression. *Cell Death Dis.* 2018;9(4):431.
