# Supplementary material for: 5‐ARI induces autophagy of prostate epithelial cells through suppressing IGF‐1 expression in prostate fibroblasts
Source: Cell Prolif. 2019 Mar 18;52(3):e12590. doi: 10.1111/cpr.12590 (PMC6536403; doi:10.1111/cpr.12590)
Supplement: Supplementary file 10 [file CPR-52-e12590-s010.docx]

**Supplementary Figure Legends**

**Supplementary Figure 1** Result of qRT-PCR array. Statistical graph of 2.0-fold up- or downregulated genes.

**Supplementary Figure 2** AR signalling impairment reduces IGF-1 expression in prostate primary fibroblasts and induces autophagy in co-cultured prostate epithelial cells. The prostate primary fibroblasts were treated with 0 nM, 1 nM or 10 nM DHT for 24h (A) The expression of IGF-1 were analysed by qRT-PCR (B) The levels of IGF-1 and AR were detected by Western blotting. (C-E) The supernatant of prostate primary fibroblasts was collected and incubated with BPH-1 cells for 2h. (C) *Left*, western blotting analysis of p-mTOR, mTOR, p62, Beclin-1, Atg5 and LC3-II. *Right*, Quantification of relative LC3-II expression. (D) TEM analysis for detecting autophagic vacuoles in BPH-1 cultured with the supernatant of prostate primary fibroblasts for 1 hour before fixation (scale bars: 2 μm). (E) Quantification of the number of autophagic vacuoles. *P<0.05, ** P<0.01, ***P<0.001

**Supplementary Figure 3** Autophagy regulates apoptosis in prostate epithelial cells. (A) Flow cytometry detected BPH-1 apoptosis after 50 nM RAPA or 50 μM CQ or control treatment for different time. RAPA-: DMSO as control group; CQ-: ddH2O as control group. (B) EdU kits were used to analyse proliferation of BPH-1 cells treated with 100 ng/ml IGF-1 or ddH_2_O for 24 h (scale bars: 50 μm). (C) Quantification of the EdU positive cells proportion. *P<0.05, ** P<0.01, ***P<0.001

**Supplementary Figure 4** Androgen signalling impairment induces autophagy in prostatic stromal cells. WPMY-1-AR cells were treated with 0 nM, 1 nM or 10 nM DHT for 24h following incubation with 50 nM RAPA or 50 μM CQ for 3 hours.(A) Western blotting results of p-mTOR, mTOR, p62, beclin-1, Atg5 and LC3-II (B). (C)

Intracellular fluorescence of mRFP-GFP-LC3 with confocal laser scanning microscopy. (scale bars: 10 μm). Veh: vehicle control group. (D) Quantification of autophagosomes (yellow) and autolysosomes (red). (E and F) Transmission electron microscopy and statistical analysis for detecting autophagic vacuoles in WPMY-1-AR (scale bars: 2 μm). *P<0.05, ** P<0.01, ***P<0.001

**Supplementary Table 1** Result of qRT-PCR array.

**Supplementary Table 2** Clinical parameters of the participants in this study.

**Supplementary Table 3** Statistical results of the number of people with different Immunohistochemical staining scores of IGF-1, SNCA, TNF-α, CXCR4 and IFNG expression in prostate epithelium of each group of patients.

**Supplementary Table 4** Statistical results of the number of people with different Immunohistochemical staining scores of LC3 and Beclin-1 expression in prostate epithelium of each group of patients.
